# Asymmetric Tear Secretion: Can This Disorder Help in Suspecting Gastroesophageal Reflux Disease and in Managing Sjögren’s Disease? A Pilot Study

**DOI:** 10.3390/medicina62010176

**Published:** 2026-01-15

**Authors:** Vilius Kontenis, Jūratė Gruodė, Jurgita Urbonienė, Almantas Šiaurys, Diana Mieliauskaitė

**Affiliations:** Department of Personalized Medicine, State Research Institute Center for Innovative Medicine, Santariskiu St. 5, LT-08406 Vilnius, Lithuania; jurate.gruode@gmail.com (J.G.); jurgita.urboniene@gmail.com (J.U.); almantas.siaurys@imcentras.lt (A.Š.); diana.mieliauskaite@imcentras.lt (D.M.)

**Keywords:** Sjögren’s disease, asymmetric tear secretion, ocular dryness, gastroesophageal reflux disease, non-autoimmune sicca syndrome, *Helicobacter pylori*

## Abstract

*Background and Objectives*: Patients with Sjögren’s disease (SjD) do not experience any improvement in gastroesophageal reflux disease (GERD) symptoms after SjD treatment, and in some patients, reflux even worsens. It is important to note that GERD manifests itself through typical and atypical symptoms, the latter of which may include eye damage, as evidenced by a growing body of research. When SjD patients were prescribed medication to treat GERD, their condition improved at the same time. Therefore, we aim to investigate whether there is a link between ocular dryness and gastroesophageal reflux disease (GERD) in patients with Sjögren’s disease (SjD). *Materials and Methods*: Our study included 27 patients with SjD according to the 2016 American College of Rheumatology and the European League Against Rheumatism (ACR/EULAR) Sjögren’s syndrome Classification Criteria, and 28 patients with non-autoimmune sicca syndrome due to GERD (nonautoimmSicca). *Results*: The study involved 55 participants, 48 (87.3%) women and 7 (12.7%) men. The median age was 54 years (IQR 49–64). A total of 41 subjects (74.5%) had GERD, and 20 subjects (36.4%) tested positive for *Helicobacter pylori*: 13 (48.1%) and 1 (3.7%) in the SjD group, and 28 (100.0%) and 19 (67.9%) in the nonautoimmSicca group, respectively. A significant difference in asymmetric tear secretion (*p* < 0.001) was found between the nonautoimmSicca and SjD patients, with values of 5 (3–10) mm/5 min and 1 (0–2) mm/5 min, respectively. A low correlation was detected between sialometry results and tear secretion asymmetry (r = 0.48, *p* < 0.001). An increase of 1 mm/5 min in the tear secretion asymmetry between the eyes was associated with a 2.04-fold increase in the odds ratio for having GERD (95% CI 1.25–3.32, *p* = 0.004), and was associated with a 1.9-fold increase in the odds ratio for having GERD (95% CI 1.04–3.49, *p* = 0.038) in patients with SjD. The presence of *Helicobacter pylori* is associated with asymmetric tear secretion [95% CI 1.22 (1.05–1.41, *p* = 0.010)]. *Conclusions*: Asymmetric tear secretion between the eyes is associated with the odds of having GERD. Patients with non-autoimmune sicca syndrome due to GERD have significantly greater asymmetry in tear secretion compared to those diagnosed with Sjögren’s disease.

## 1. Introduction

Gastroesophageal reflux disease (GERD) occurs when stomach contents flow back into the esophagus and beyond, causing symptoms, mucosal damage, and long-term complications. The prevalence of GERD is increasing worldwide and is estimated to affect 8–33% of the global population. GERD posits a significant global health challenge [1,2,3].

Recent research findings show a genetic link between GERD and Sjögren’s disease (SjD) and that GERD is a risk factor for Sjögren’s disease, while SjD does not affect GERD. It has been observed that patients with Sjögren’s disease did not experience any improvement in GERD symptoms after SjD treatment, and in some patients, reflux worsened due to the side effects of the medication. Strangely and interestingly, when SjD patients were prescribed medication to treat GERD, their condition improved at the same time [4,5].

Sjögren’s disease (SjD) is the prototype of a local and systemic autoimmune disease caused by B-cell hyperreactivity and characterised by exocrine gland dysfunction and various systemic manifestations, and an increased risk of lymphoma [6].

The prevalence of extraglandular manifestations (EGMs) in SjD has been reported to range from 30% to 90%, depending on how EGMs are defined and the size of the cohort assessed. Extraglandular manifestations in Sjögren’s disease (SjD) represent the clinical expression of the systemic involvement in this disease. EGMs are characterized by a wide heterogeneity; virtually any organ or system can be affected, with various degrees of dysfunction [7,8]. The EULAR Sjögren’s Syndrome (SS) Disease Activity Index (ESSDAI) is a systemic disease activity index designed to measure disease activity in patients with Sjögren’s disease. ESSDAI is now used as the gold standard for assessing disease activity in clinical trials, and as a baseline, even a core indicator, in current randomised clinical trials. It consists of 12 domains (cutaneous, pulmonary, renal, articular, muscular, peripheral nervous system (PNS), central nervous system (CNS), haematological, glandular, constitutional, lymphadenopathy and lymphoma, biological) [8,9,10]. Although EULAR Sjögren’s Syndrome (SS) Disease Activity Index does not include the gastrointestinal tract, the main gastrointestinal manifestations of Sjögren’s disease can occur in the oesophagus, stomach, pancreas, liver and small intestine. Gastroesophageal reflux (GER) is present in 13 to 60% of SjD patients [7]. It is important to note that in recent years, there has been growing evidence that GERD also affects the surface of the eye [11]. This undoubtedly further reduces quality of life, especially for those suffering from other diseases, such as Sjögren’s disease, which also affects the eyes. The results of a recently published Mendelian randomization analysis of two samples show a clear association between *Helicobacter pylori* (*H. pylori*) IgG antibody status and enhanced predisposition to GERD [12]. It is also important to note that the results of the Mendelian randomization analysis confirm the causal link between H. pylori infection and SjD and show that SjD may increase the risk of *H. pylori* infection [13]. *H. pylori* is a chronic pathogen that colonizes the stomach epithelium and affects 30–50% of people in developed countries and as many as 80% of people in developing countries [14]. *H. pylori* is the main cause of peptic ulcers and gastric cancer, and there is growing evidence of a link between *H. pylori* and extra-gastric pathologies [15]. All of this encourages the search for clinical signs that would allow early suspicion and diagnosis of GERD in patients with SjD.

Objectives: To investigate whether there is a link between ocular dryness and gastroesophageal reflux disease (GERD) in patients with Sjögren’s disease (SjD).

## 2. Materials and Methods

### 2.1. Study Groups and Groups Formation

The study included patients who had been examined between September 2024 and October 2025 at the outpatient department of the Innovative Medicine Centre of the State Research Institute (Vilnius, Lithuania) to investigate the cause of subjective dry mouth and/or dry eye symptoms. The diagnosis of Sjögren’s disease and non-autoimmune sicca syndrome due to GERD was firstly established before enrolling in this study. Our study included age-matched 27 patients with Sjögren’s disease according to the 2016 American College of Rheumatology (ACR) and the European League Against Rheumatism (now ‘European Alliance of Associations for Rheumatology’) (ACR/EULAR) Sjögren’s syndrome classification criteria [10] and 28 patients with non-autoimmune sicca syndrome due to GERD as a comparative group (Table 1).

Sjögren’s disease was diagnosed based on the weighted sum of 5 elements: anti-SSA (Ro) antibody positivity, 3 points; focal lymphocytic sialadenitis with focal scores ≥ 1 focus/mm^2^, 3 points; abnormal eye staining score ≥ 5 using fluorescein, 1 point; Schirmer test ≤ 5 mm/5 min, 1 point, and unstimulated salivary flow ≤0.1 mL/min, 1 point. Individuals (with SjD signs/symptoms) whose total score, based on the above criteria, was ≥4, met the diagnosis of Sjögren’s disease.

The comparative group of non-autoimmune sicca syndrome included individuals who had been diagnosed with GERD for the first time and who complained of dry eye and/or mouth symptoms (atypical symptoms). The subjects were diagnosed with GERD by a licensed gastroenterologist-endoscopist based on clinical signs of GERD and the results of esophagogastroduodenoscopy at the Institute’s outpatient department. These patients were examined for signs of ocular and oral dryness, anti-SSA(Ro) antibody positivity and focal lymphocytic sialadenitis with focal scores ≥ 1 focus/mm^2^. The subjects of this group did not meet the criteria for Sjögren’s disease, and no other possible causes of dry eyes and mouth other than GERD were found in these subjects.

*Helicobacter pylori* was detected in stool samples using the *Helicobacter pylori* stool antigen (HpSA) test by ELISA.

All subjects underwent the Schirmer test during morning working hours (from 8:00 to 10:00 a.m.). The subjects did not use any eye drops (medications, artificial tears) for 12 h prior to the test, and none of the subjects of the study ever wore contact lenses.

Performing unstimulated sialometry involves preparing the patient (no eating/drinking, empty mouth for 2 h before the test), positioning (sitting, head slightly tilted forward), collection (15 min of saliva in a special test tube, do not swallow) and measurement (total volume in mL/15 min). Test procedure includes: swallow saliva, tilt head forward, collect saliva in a tube/funnel for 15 min without stimulating the glands, measure the total volume.

Patients in both target groups of the study had not yet started treatment for either Sjögren’s disease or GERD.

### 2.2. Ethical Considerations

This study protocol was approved by the Vilnius Regional Biomedical Research Ethics Committee (20 December 2022, No. 2022/12-1485-949) and was in accordance with the tenets set forth in the Declaration of Helsinki. Written informed consent was obtained from participants prior to participation in this study.

### 2.3. Statistical Analysis

Continuous and categorical variables were presented as median interquartile range (IQR) and numbers (percentages, %), respectively. The Mann–Whitney U test was used to compare continuous variables, and χ^2^ test or Fisher exact test was used to compare categorical variables. Spearman correlation was used to investigate the correlation between continuous variables. The strength of correlation was interpreted as follows: r value 0.1 to 0.3 negligible correlation, 0.3 to 0.5 low correlation, 0.5 to 0.7 moderate, 0.7 to 0.9 strong correlation, and 0.9 to 1.0 very strong correlation.

Two univariable binary logistic regression models were created to determine the association of Schirmer’s test results, unstimulated sialometry results, and the presence of GERD or positive *Helicobacter pylori*. The first model included the presence of GERD as the dependent variable, with age, Schirmer’s test results in each eye, interocular Schirmer’s test difference, and unstimulated sialometry results as predictors. The second model included the presence of *H. pylori* as the dependent variable, with the same predictors. Version number of the software used IBM SPSS Statistics 30.0. A two-sided *p*-value < 0.05 was considered significant.

## 3. Results

The study involved 55 participants, 48 (87.3%) women and 7 (12.7%) men. The median age was 54 years (IQR 49–64). A total of 41 subjects (74.5%) had GERD, and 20 subjects (36.4%) tested positive for *H. pylori*: 28 (100.0%) and 19 (67.9%) in the nonautoimmSicca group, and 13 (48.1%) and 1 (3.7%) in the SjD group, respectively. Significant asymmetric tear secretion between the eyes (*p* < 0.001) was found between the nonautoimmSicca and SjD patients according to Schirmer’s test, with values of 5 (3–10) mm/5 min and 1 (0–2) mm/5 min, respectively. A significant difference was found in total saliva production by unstimulated sialometry (mL/15 min) between nonautoimmSicca and SjD patients, with values of 2.85 (1.23–4.38) and 1 (0.7–1.3), respectively. A low correlation was detected between unstimulated sialometry results and asymmetric tear secretion between the eyes according to Schirmer’s test results (r = 0.48, *p* < 0.001). An increase in tear secretion asymmetry between the eyes of 1 mm/5 min was associated with a 2.04-fold increase in the odds ratio for having GERD (95% CI 1.25–3.32, *p* = 0.004). Similarly, an increase of 1 mL/15 min in unstimulated sialometry results was associated with a 2.17-fold increase in the odds ratio for having GERD (95% CI 1.06–4.43, *p* = 0.033). An increase of 1 mm/5 min in tear secretion asymmetry between the eyes was associated with a 1.9-fold increase in the odds ratio for having GERD (95% CI 1.04–3.49, *p* = 0.038) in patients with SjD. We found that the presence of *H. pylori* is associated with tear secretion asymmetry between the eyes according to Schirmer’s test results (95% CI 1.22 (1.05–1.41, *p* = 0.010) (Table 2, Table 3 and Table 4).

## 4. Discussion

Our empirical clinical observation that asymmetric tear secretion between the eyes leads to the diagnosis of GERD led to the evaluation of a link between ocular dryness expression and gastroesophageal reflux disease in patients with Sjögren’s disease.

GERD presents with a wide range of symptoms, from mild, intermittent episodes to severe, daily episodes that have a significant impact on quality of life [16]. Recent research findings show a genetic link between GERD and Sjögren’s disease (SjD), and that GERD is a risk factor for SjD, while SjD does not affect GERD. The results of these studies encourage greater attention to be paid to the potential consequences of GERD [5]. It is important to note that GERD manifests itself through various symptoms, which are divided into two groups: typical (heartburn, regurgitation, and non-cardiac chest pain) and atypical or non-esophageal (asthma, chronic cough, laryngitis, hoarseness, persistent sore throat, and damage to tooth enamel) [3,17].

SjD is one of the autoimmune diseases with the maximum delay in diagnosis due to its insidious onset, heterogeneous clinical features and varied course. In addition, SjD diagnosis is complicated by the variety of symptoms, which means that the correct diagnosis is made later (often after many years). To date, there is no specific treatment for Sjögren’s disease, so treatment is limited to alleviating the symptoms [6,18,19]. So, it is increasingly recognized that extraglandular manifestations represent a clinical challenge for patients with SjD [7,8].

Our pilot study showed a significantly different asymmetry in tear secretion between patients with Sjögren’s disease and non-autoimmune sicca syndrome due to GERD. In this study, we did not aim to elucidate the pathogenetic mechanisms of tear secretion asymmetry and attempted to explain it based on the results of the latest scientific research. We believe that autoimmune inflammation disrupts tear secretion in both eyes to a similar degree in patients with SjD, and that the difference in tear secretion levels between the eyes is significantly smaller in patients with Sjögren’s disease compared to patients with non-autoimmune sicca syndrome caused by GERD. This is undoubtedly related to the different effects of the refluxate containing acidic fluid and pepsin on the right and left nasolacrimal ducts and lacrimal drainage system in patients with GERD, presumably due to sleeping position. As established from the medical history, the eye on the side on which the person sleeps is more affected.

A retrospective, large-sample study demonstrated that GERD is associated with an increased risk of lacrimal drainage obstruction. This study was based on findings from previous studies that the pathophysiological process of chronic sinusitis comprises the direct harmful effects of reflux on the sinus mucosa, which leads to inflammation. It has been hypothesized that GERD may play a role in the onset of lacrimal drainage obstruction because the nasolacrimal duct opens into the nasal cavity, and reflux into the nasal cavity may induce ascending inflammation that extends into the nasolacrimal duct. The researchers of this retrospective study identified the limitations of its research, stating that it was not possible to determine whether the lacrimal drainage system in both eyes was equally affected. It is likely that these researchers, like us, had in mind the effect of GERD on tear drainage depending on sleeping position [20].

The results of a recently published study show that GERD is associated with an increased risk of primary acquired nasal and tear duct obstruction, which is especially common in women, middle-aged and elderly adults, and patients with cardiovascular disease or sinusitis [21].

Eye changes associated with GERD may be caused by increased levels of the stomach enzyme pepsin. Pepsin is a stomach enzyme that is also found in the nasal cavity, sinuses, and saliva of people with GERD. Patients with laryngopharyngeal reflux (LPR) had detectable levels of pepsin in their tears, which explains the obstruction of the nasal and ocular surface. A recent study has shown that the concentration of pepsin in tears is significantly associated with the severity of LPR disease and changes in the surface of the eye [22]. GERD management options include elevating the head of the bed and sleeping on the left side among the suggested lifestyle changes [3]. Evidence suggests that for a large proportion of patients, the only GERD symptoms that manifest outside the upper gastrointestinal tract are non-upper gastrointestinal symptoms such as nasal symptoms, ear symptoms, upper respiratory tract symptoms, and autonomic (heart rhythm) and endocrine (insulin secretion) symptoms. This clinical condition, known as extraesophageal GERD (EGERD), is most commonly associated with local lesions directly caused by gastric contents [23,24]. Other studies also demonstrate a correlation between clinical reflux symptoms and ocular surface discomfort [24,25]. Several hypotheses have been offered to clarify the extraesophageal symptoms in these patients, which are most commonly linked to reverse gastric reflux. Local chronic inflammation was present in insulin-dependent type I diabetic patients in the recent study, but increases in nonspecific markers of inflammation (IL8 and HLA-DR) and oxidative stress (NADPH) were associated with parallel increases in neurotransmitters and pepsin. Local increases in neurotransmitters can cause chronic neurogenic inflammation and oxidative stress, which can lead to disorders of the ocular surface system in insulin-dependent type I diabetic patients [26]. Another recent study demonstrated that patients with laryngopharyngeal reflux (LPR) showed ocular surface changes, including epithelial damage and impairment of lacrimal function. The results of the latest study showed that patients diagnosed with dry eye disease have an eight times higher index of pathological reflux symptoms than individuals without dry eye disease [27].

Meanwhile, our research has highlighted that an increase of 1 mm/5 min in the asymmetry of tear production between the eyes according to Schirmer’s test was associated with a 2.04-fold increase in the odds ratio for having GERD. An increase of 1 mm/5 min in the asymmetry of tear production between the eyes was associated with a 1.9-fold increase in the odds ratio for having GERD in patients with SjD.

A significant difference was found in saliva production between nonautoimmSicca and SjD patients. These data indicate that autoimmune epithelitis in SjD causes a more significant impairment of salivary secretion than GERD. A low correlation was detected between sialometry results and the interocular Schirmer’s test result difference. Following the recommended lifestyle (raising the head of the bed and sleeping on the left side) will improve GERD symptoms and reduce both eye and mouth dryness [3].

We found that the presence of *H. pylori* is associated with the asymmetry of tear secretion between the eyes according to Schirmer’s test results.

Although *H. pylori* infection has long been linked to gastrointestinal diseases, its effect on otolaryngological diseases is still unclear. Recent studies show that *H. pylori* is not only present in the nasal sinus mucosa, tonsils, and throat, but also in the middle ear. Recent genetic studies have suggested a causal relationship between anti-*H. pylori* CagA levels and non-suppurative middle ear inflammation [28].

Results from a recent study show that the coexistence of *H. pylori* with oral microorganisms highlights the need to investigate the role of the oral microbiota in the cycle of *H. pylori* infection and transmission [29].

There is evidence that patients diagnosed with dry eye disease (DED) who are *H. pylori*-positive have a higher Ocular Surface Disease Index (OSDI) and more severe meibomian gland dysfunction [30].

The correlation between *H. pylori* infection and dry eye is still being investigated. The recently published results of the study investigating the effect of *Helicobacter pylori* (*H. pylori*) infection on ocular surface homeostasis have provided evidence that *H. pylori* infection impairs tear secretion and ocular surface microbiota, possibly leading to T-lymphocyte influx and lacrimal gland dysfunction, thereby contributing to the pathogenesis of dry eye [15].

A new study has revealed that changes in the surface of the eye in patients with Sjögren’s disease are significantly associated with systemic hematological indicators and disease activity [31]. Although gastrointestinal tract pathology is not assessed when determining SjD activity, we can assume that Sjögren’s disease patients diagnosed with GERD and *H. pylori*-positive experience worsening of their conditions, and it is important to diagnose GERD as early as possible.

Our study has limitations due to the sample size. However, empirical observations from our clinical work (data not published) allowed us to test the hypothesis of asymmetric tear secretion as a warning sign of gastroesophageal reflux. The study was prospective, with target groups carefully selected. We hope that the results of our pilot study will encourage both us and other researchers to conduct more in-depth studies with larger sample sizes.

The strength of our study lies in the fact that, to our knowledge, this is the first scientific evidence that GERD is characterized by tear secretion asymmetry between the eyes, which allows for early diagnosis of GERD. In addition, this will contribute to the personalized management of Sjögren’s disease, as the condition of patients with this disease improves when treated for gastroesophageal reflux disease. The study is being continued with the analysis of a larger sample of oral microbiota and clinical manifestations in patients with Sjögren’s disease and non-autoimmune sicca syndrome due to GERD.

## 5. Conclusions

Patients with non-autoimmune sicca syndrome due to GERD have significantly greater asymmetry in tear secretion compared to those diagnosed with Sjögren’s disease. Asymmetric tear secretion between the eyes is associated with increased odds of having GERD.

## Figures and Tables

**Table 1:** Demographic and clinical characteristics of patients with non-autoimmune sicca syndrome due to gastroesophageal reflux disease and Sjögren’s disease.

Variable	Non-Autoimmune Sicca Syndrome, n = 28	Sjögren‘s Disease, n = 27	*p*
Age, years	52.5 (45.5–63.75)	54 (49–64)	0.553
Female, n (%)	21 (75.0)	27 (100.0)	0.010
Patients with GERD, n (%)	28 (100.0)	13 (48.1)	<0.001
Positive *H. pylori*, n (%)	19 (67.9)	1 (3.7)	<0.001
Asymmetric tear secretion, interocular Schirmer’s test difference, mm/5 min	5 (3–10)	1 (0–2)	<0.001
Unstimulated sialometry, total volume, mL/15 min	2.85 (1.23–4.38)	1 (0.7–1.3)	<0.001

GERD = Gastroesophageal reflux disease; *H. pylori* = *Helicobacter pylori.*

**Table 2:** The relationship between gastroesophageal reflux disease and asymmetric tear secretion and saliva secretion.

Variable	n	Crude OR, 95% CI	*p*
Age, years	55	0.99 (0.93–1.05)	0.695
Asymmetric tear secretion, interocular Schirmer’s test difference, mm/5 min	55	2.04 (1.25–3.32)	0.004
Unstimulated sialometry, total volume, mL/15 min	55	2.17 (1.06–4.43)	0.033

**Table 3:** The relationship between gastroesophageal reflux disease and asymmetric tear secretion and saliva secretion in patients with Sjögren’s disease.

Variable	n	Crude OR, 95% CI	*p*
Age, years	27	0.99 (0.92–1.07)	0.820
Asymmetric tear secretion, interocular Schirmer’s test difference, mm/5 min	27	1.90 (1.04–3.49)	0.038
Unstimulated sialometry, total volume, mL/15 min	27	0.82 (0.27–2.50)	0.724

**Table 4:** The relationship between *Helicobacter pylori* presence and tear secretion and saliva secretion.

Variable	n	Crude OR, 95% CI	*p*
Age, years	55	0.98 (0.93–1.03)	0.451
Asymmetric tear secretion, interocular Schirmer’s test difference, mm/5 min	55	1.22 (1.05–1.41)	0.010
Unstimulated sialometry, total volume, mL/15 min	55	1.28 (0.89–1.84)	0.186

## Data Availability

The data presented in this study are available on request from the corresponding author.
